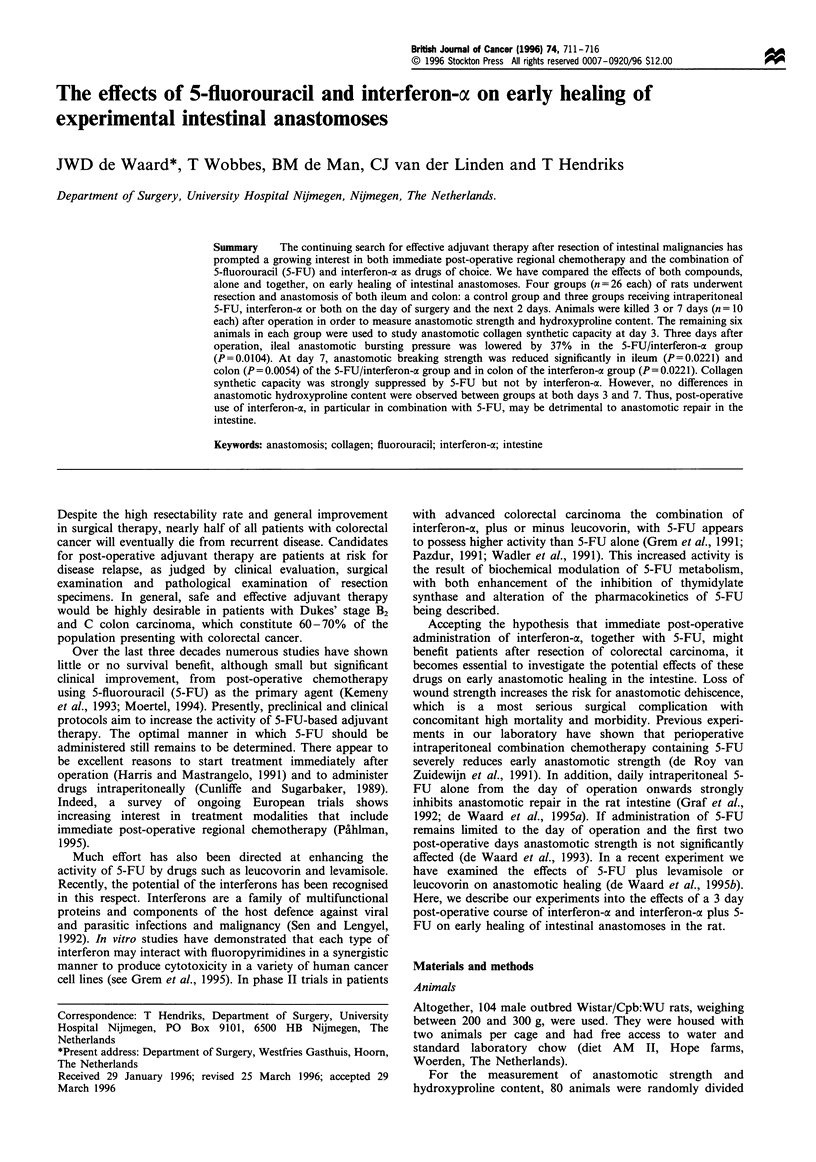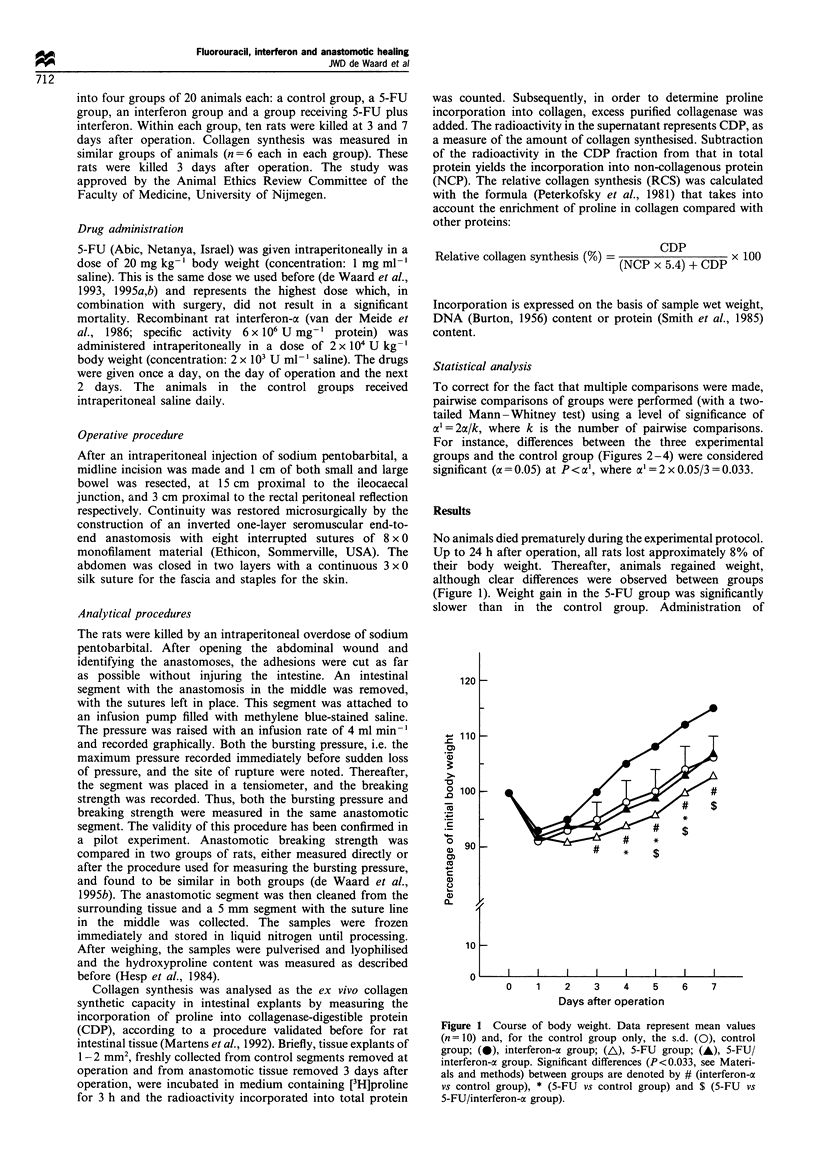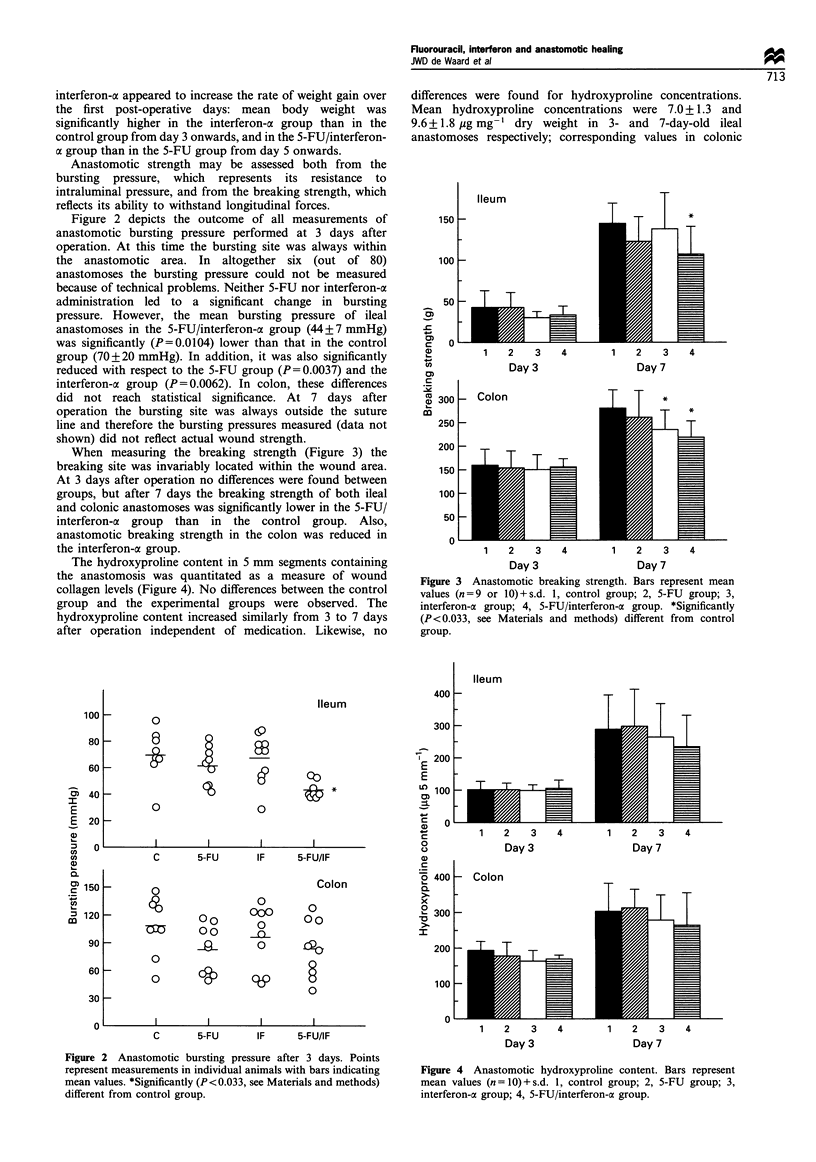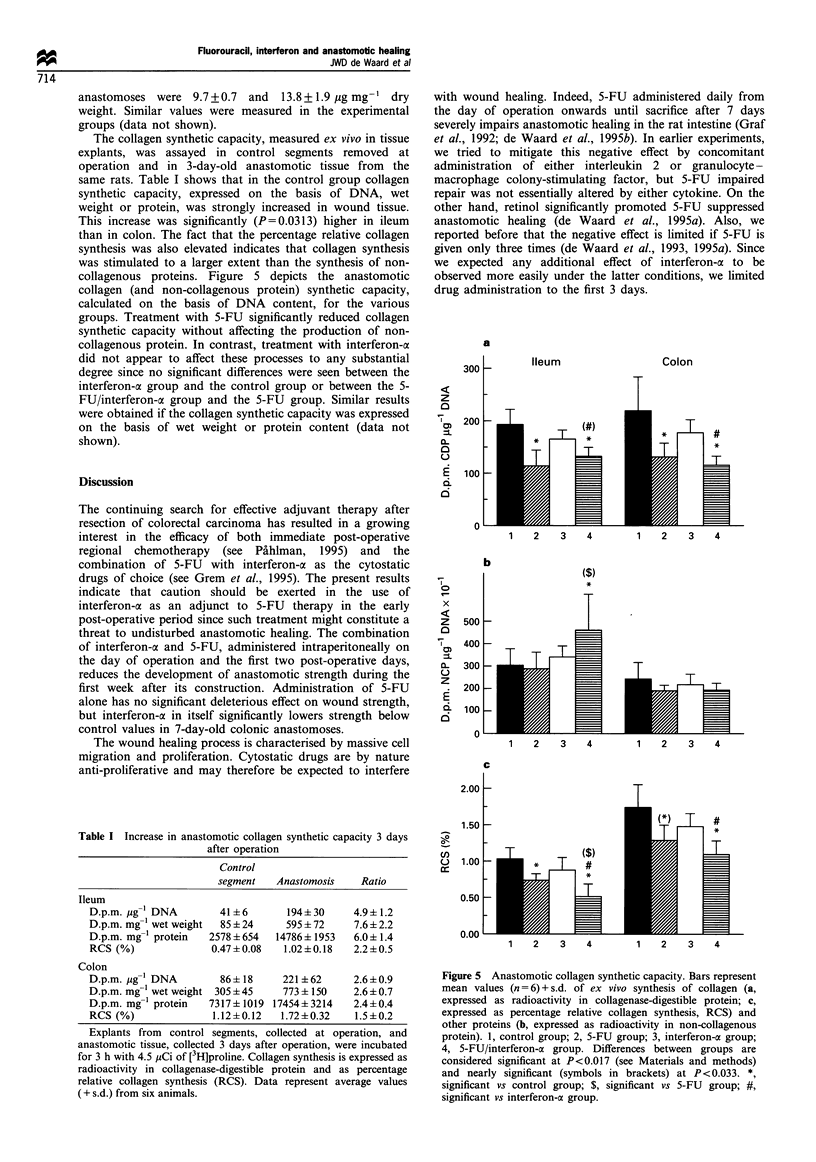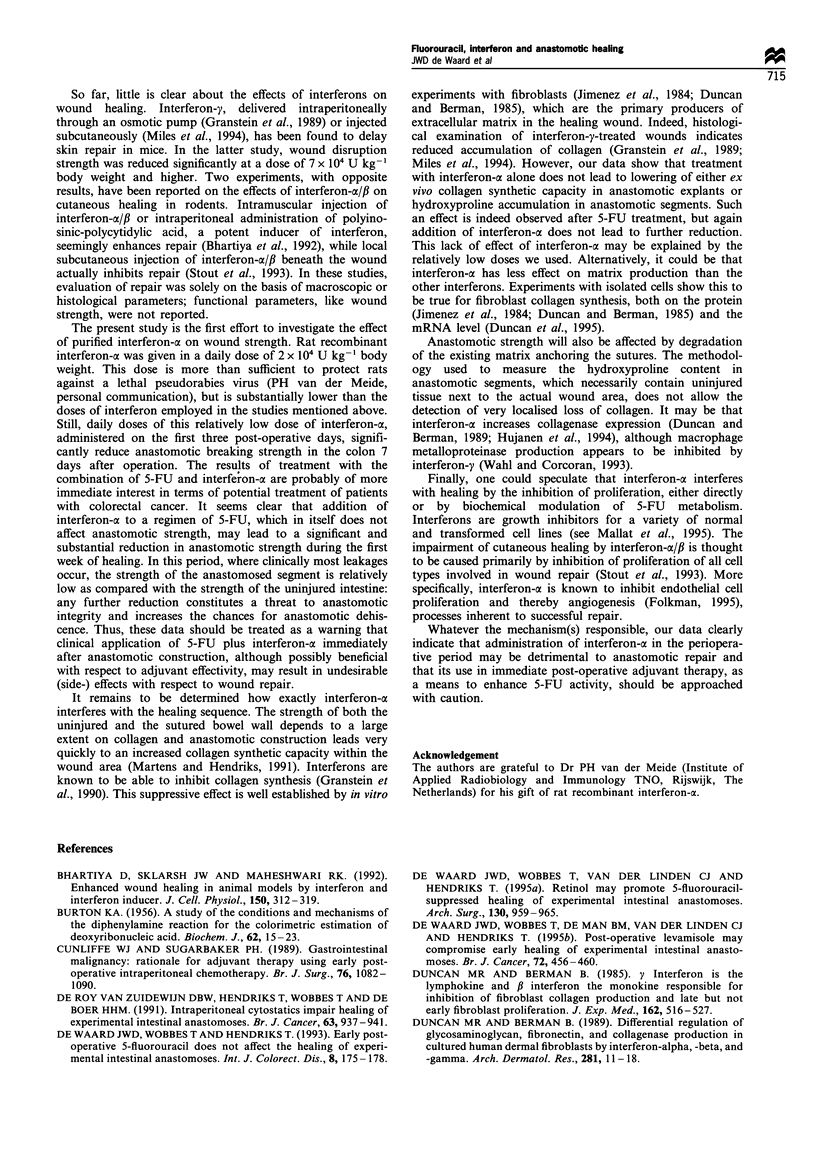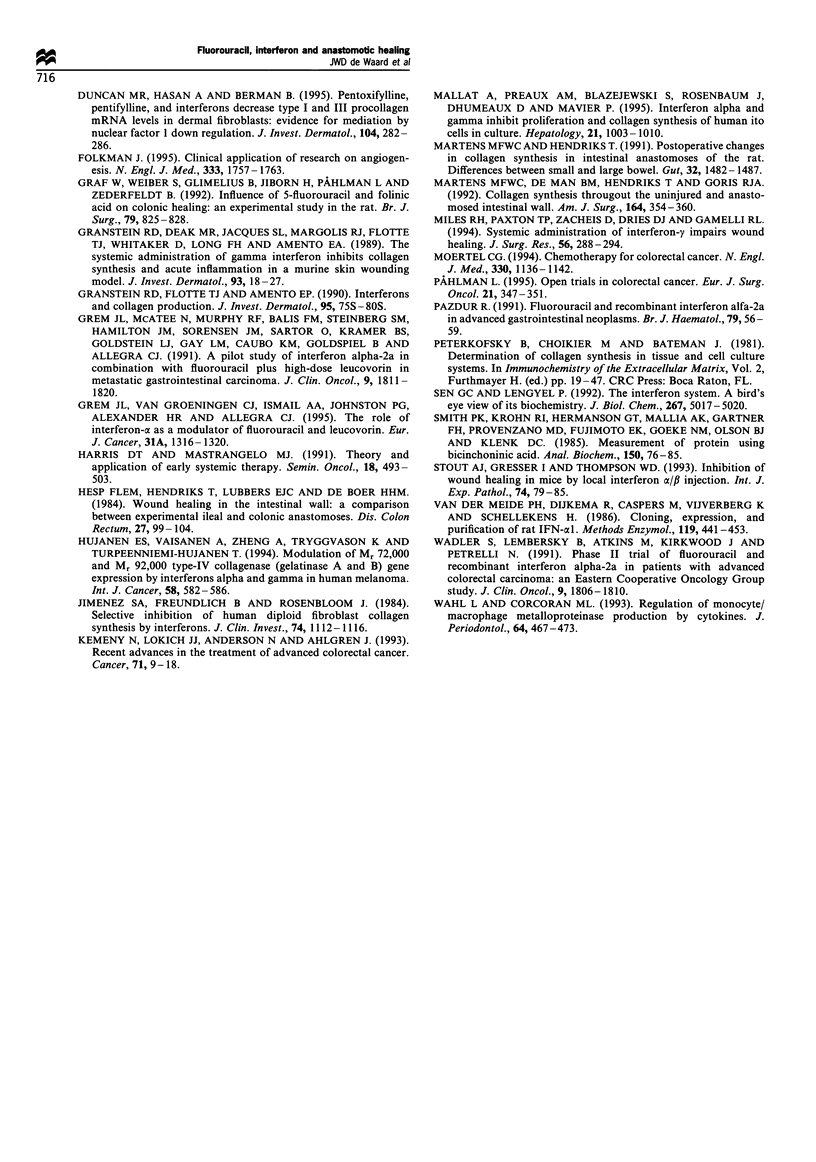# The effects of 5-fluorouracil and interferon-alpha on early healing of experimental intestinal anastomoses.

**DOI:** 10.1038/bjc.1996.426

**Published:** 1996-09

**Authors:** J. W. de Waard, T. Wobbes, B. M. de Man, C. J. van der Linden, T. Hendriks

**Affiliations:** Department of Surgery, University Hospital Nijmegen, The Netherlands.

## Abstract

The continuing search for effective adjuvant therapy after resection of intestinal malignancies has prompted a growing interest in both immediate post-operative regional chemotherapy and the combination of 5-fluorouracil (5-FU) and interferon-alpha as drugs of choice. We have compared the effects of both compounds, alone and together, on early healing of intestinal anastomoses. Four groups (n = 26 each) of rats underwent resection and anastomosis of both ileum and colon: a control group and three groups receiving intraperitoneal 5-FU, interferon-alpha or both on the day of surgery and the next 2 days. Animals were killed 3 or 7 days (n = 10 each) after operation in order to measure anastomotic strength and hydroxyproline content. The remaining six animals in each group were used to study anastomotic collagen synthetic capacity at day 3. Three days after operation, ileal anastomotic bursting pressure was lowered by 37% in the 5-FU/interferon-alpha group (P = 0.0104). At day 7, anastomotic breaking strength was reduced significantly in ileum (P = 0.0221) and colon (P = 0.0054) of the 5-FU/interferon-alpha group and in colon of the interferon-alpha group (P = 0.0221). Collagen synthetic capacity was strongly suppressed by 5-FU but not by interferon-alpha. However, no differences in anastomotic hydroxyproline content were observed between groups at both days 3 and 7. Thus, post-operative use of interferon-alpha, in particular in combination with 5-FU, may be detrimental to anastomotic repair in the intestine.


British Journal of Cancer (1996) 74, 711-716
© 1996 Stockton Press All rights reserved 0007-0920/96 $12.00

### The effects of 5-fluorouracil and interferon-α on early healing of experimental intestinal anastomoses

JWD de Waard*, T Wobbes, BM de Man, CJ van der Linden and T Hendriks

*Department of Surgery, University Hospital Nijmegen, Nijmegen, The Netherlands.*

**Summary** The continuing search for effective adjuvant therapy after resection of intestinal malignancies has prompted a growing interest in both immediate post-operative regional chemotherapy and the combination of 5-fluorouracil (5-FU) and interferon-α as drugs of choice. We have compared the effects of both compounds, alone and together, on early healing of intestinal anastomoses. Four groups ($n=26$ each) of rats underwent resection and anastomosis of both ileum and colon: a control group and three groups receiving intraperitoneal 5-FU, interferon-α or both on the day of surgery and the next 2 days. Animals were killed 3 or 7 days ($n=10$ each) after operation in order to measure anastomotic strength and hydroxyproline content. The remaining six animals in each group were used to study anastomotic collagen synthetic capacity at day 3. Three days after operation, ileal anastomotic bursting pressure was lowered by 37% in the 5-FU/interferon-α group ($P=0.0104$). At day 7, anastomotic breaking strength was reduced significantly in ileum ($P=0.0221$) and colon ($P=0.0054$) of the 5-FU/interferon-α group and in colon of the interferon-α group ($P=0.0221$). Collagen synthetic capacity was strongly suppressed by 5-FU but not by interferon-α. However, no differences in anastomotic hydroxyproline content were observed between groups at both days 3 and 7. Thus, post-operative use of interferon-α, in particular in combination with 5-FU, may be detrimental to anastomotic repair in the intestine.

**Keywords:** anastomosis; collagen; fluorouracil; interferon-α; intestine

Despite the high resectability rate and general improvement in surgical therapy, nearly half of all patients with colorectal cancer will eventually die from recurrent disease. Candidates for post-operative adjuvant therapy are patients at risk for disease relapse, as judged by clinical evaluation, surgical examination and pathological examination of resection specimens. In general, safe and effective adjuvant therapy would be highly desirable in patients with Dukes' stage $B_2$ and C colon carcinoma, which constitute 60-70% of the population presenting with colorectal cancer.

Over the last three decades numerous studies have shown little or no survival benefit, although small but significant clinical improvement, from post-operative chemotherapy using 5-fluorouracil (5-FU) as the primary agent (Kemeny *et al.*, 1993; Moertel, 1994). Presently, preclinical and clinical protocols aim to increase the activity of 5-FU-based adjuvant therapy. The optimal manner in which 5-FU should be administered still remains to be determined. There appear to be excellent reasons to start treatment immediately after operation (Harris and Mastrangelo, 1991) and to administer drugs intraperitoneally (Cunliffe and Sugarbaker, 1989). Indeed, a survey of ongoing European trials shows increasing interest in treatment modalities that include immediate post-operative regional chemotherapy (Påhlman, 1995).

Much effort has also been directed at enhancing the activity of 5-FU by drugs such as leucovorin and levamisole. Recently, the potential of the interferons has been recognised in this respect. Interferons are a family of multifunctional proteins and components of the host defence against viral and parasitic infections and malignancy (Sen and Lengyel, 1992). *In vitro* studies have demonstrated that each type of interferon may interact with fluoropyrimidines in a synergistic manner to produce cytotoxicity in a variety of human cancer cell lines (see Grem *et al.*, 1995). In phase II trials in patients with advanced colorectal carcinoma the combination of interferon-α, plus or minus leucovorin, with 5-FU appears to possess higher activity than 5-FU alone (Grem *et al.*, 1991; Pazdur, 1991; Wadler *et al.*, 1991). This increased activity is the result of biochemical modulation of 5-FU metabolism, with both enhancement of the inhibition of thymidylate synthase and alteration of the pharmacokinetics of 5-FU being described.

Accepting the hypothesis that immediate post-operative administration of interferon-α, together with 5-FU, might benefit patients after resection of colorectal carcinoma, it becomes essential to investigate the potential effects of these drugs on early anastomotic healing in the intestine. Loss of wound strength increases the risk for anastomotic dehiscence, which is a most serious surgical complication with concomitant high mortality and morbidity. Previous experiments in our laboratory have shown that perioperative intraperitoneal combination chemotherapy containing 5-FU severely reduces early anastomotic strength (de Roy van Zuidewijn *et al.*, 1991). In addition, daily intraperitoneal 5-FU alone from the day of operation onwards strongly inhibits anastomotic repair in the rat intestine (Graf *et al.*, 1992; de Waard *et al.*, 1995*a*). If administration of 5-FU remains limited to the day of operation and the first two post-operative days anastomotic strength is not significantly affected (de Waard *et al.*, 1993). In a recent experiment we have examined the effects of 5-FU plus levamisole or leucovorin on anastomotic healing (de Waard *et al.*, 1995*b*). Here, we describe our experiments into the effects of a 3 day post-operative course of interferon-α and interferon-α plus 5-FU on early healing of intestinal anastomoses in the rat.

### Materials and methods

### *Animals*

Altogether, 104 male outbred Wistar/Cpb:WU rats, weighing between 200 and 300 g, were used. They were housed with two animals per cage and had free access to water and standard laboratory chow (diet AM II, Hope farms, Woerden, The Netherlands).

For the measurement of anastomotic strength and hydroxyproline content, 80 animals were randomly divided

Correspondence: T Hendriks, Department of Surgery, University Hospital Nijmegen, PO Box 9101, 6500 HB Nijmegen, The Netherlands
*Present address: Department of Surgery, Westfries Gasthuis, Hoorn, The Netherlands
Received 29 January 1996; revised 25 March 1996; accepted 29 March 1996

into four groups of 20 animals each: a control group, a 5-FU group, an interferon group and a group receiving 5-FU plus interferon. Within each group, ten rats were killed at 3 and 7 days after operation. Collagen synthesis was measured in similar groups of animals ($n=6$ each in each group). These rats were killed 3 days after operation. The study was approved by the Animal Ethics Review Committee of the Faculty of Medicine, University of Nijmegen.

*Drug administration*

5-FU (Abic, Netanya, Israel) was given intraperitoneally in a dose of 20 mg $kg^{-1}$ body weight (concentration: 1 mg $ml^{-1}$ saline). This is the same dose we used before (de Waard *et al.*, 1993, 1995*a*,*b*) and represents the highest dose which, in combination with surgery, did not result in a significant mortality. Recombinant rat interferon-α (van der Meide *et al.*, 1986; specific activity $6\times10^{6}$ U $mg^{-1}$ protein) was administered intraperitoneally in a dose of $2\times10^{4}$ U $kg^{-1}$ body weight (concentration: $2\times10^{3}$ U $ml^{-1}$ saline). The drugs were given once a day, on the day of operation and the next 2 days. The animals in the control groups received intraperitoneal saline daily.

*Operative procedure*

After an intraperitoneal injection of sodium pentobarbital, a midline incision was made and 1 cm of both small and large bowel was resected, at 15 cm proximal to the ileocaecal junction, and 3 cm proximal to the rectal peritoneal reflection respectively. Continuity was restored microsurgically by the construction of an inverted one-layer seromuscular end-to-end anastomosis with eight interrupted sutures of 8×0 monofilament material (Ethicon, Sommerville, USA). The abdomen was closed in two layers with a continuous 3×0 silk suture for the fascia and staples for the skin.

*Analytical procedures*

The rats were killed by an intraperitoneal overdose of sodium pentobarbital. After opening the abdominal wound and identifying the anastomoses, the adhesions were cut as far as possible without injuring the intestine. An intestinal segment with the anastomosis in the middle was removed, with the sutures left in place. This segment was attached to an infusion pump filled with methylene blue-stained saline. The pressure was raised with an infusion rate of 4 ml $min^{-1}$ and recorded graphically. Both the bursting pressure, i.e. the maximum pressure recorded immediately before sudden loss of pressure, and the site of rupture were noted. Thereafter, the segment was placed in a tensiometer, and the breaking strength was recorded. Thus, both the bursting pressure and breaking strength were measured in the same anastomotic segment. The validity of this procedure has been confirmed in a pilot experiment. Anastomotic breaking strength was compared in two groups of rats, either measured directly or after the procedure used for measuring the bursting pressure, and found to be similar in both groups (de Waard *et al.*, 1995*b*). The anastomotic segment was then cleaned from the surrounding tissue and a 5 mm segment with the suture line in the middle was collected. The samples were frozen immediately and stored in liquid nitrogen until processing. After weighing, the samples were pulverised and lyophilised and the hydroxyproline content was measured as described before (Hesp *et al.*, 1984).

Collagen synthesis was analysed as the *ex vivo* collagen synthetic capacity in intestinal explants by measuring the incorporation of proline into collagenase-digestible protein (CDP), according to a procedure validated before for rat intestinal tissue (Martens *et al.*, 1992). Briefly, tissue explants of 1–2 $mm^2$, freshly collected from control segments removed at operation and from anastomotic tissue removed 3 days after operation, were incubated in medium containing [$^3$H]proline for 3 h and the radioactivity incorporated into total protein was counted. Subsequently, in order to determine proline incorporation into collagen, excess purified collagenase was added. The radioactivity in the supernatant represents CDP, as a measure of the amount of collagen synthesised. Subtraction of the radioactivity in the CDP fraction from that in total protein yields the incorporation into non-collagenous protein (NCP). The relative collagen synthesis (RCS) was calculated with the formula (Peterkofsky *et al.*, 1981) that takes into account the enrichment of proline in collagen compared with other proteins:

$$\text{Relative collagen synthesis (\%)} = \frac{\text{CDP}}{(\text{NCP}\times 5.4)+\text{CDP}}\times 100$$

Incorporation is expressed on the basis of sample wet weight, DNA (Burton, 1956) content or protein (Smith *et al.*, 1985) content.

*Statistical analysis*

To correct for the fact that multiple comparisons were made, pairwise comparisons of groups were performed (with a two-tailed Mann–Whitney test) using a level of significance of $\alpha^1=2\alpha/k$, where $k$ is the number of pairwise comparisons. For instance, differences between the three experimental groups and the control group (Figures 2–4) were considered significant ($\alpha=0.05$) at $P<\alpha^1$, where $\alpha^1=2\times0.05/3=0.033$.

### Results

No animals died prematurely during the experimental protocol. Up to 24 h after operation, all rats lost approximately 8% of their body weight. Thereafter, animals regained weight, although clear differences were observed between groups (Figure 1). Weight gain in the 5-FU group was significantly slower than in the control group. Administration of

**Figure 1** Course of body weight. Data represent mean values ($n=10$) and, for the control group only, the s.d. (○), control group; (●), interferon-α group; (△), 5-FU group; (▲), 5-FU/interferon-α group. Significant differences ($P<0.033$, see Materials and methods) between groups are denoted by # (interferon-α *vs* control group), * (5-FU *vs* control group) and $ (5-FU *vs* 5-FU/interferon-α group).

interferon-α appeared to increase the rate of weight gain over the first post-operative days: mean body weight was significantly higher in the interferon-α group than in the control group from day 3 onwards, and in the 5-FU/interferon-α group than in the 5-FU group from day 5 onwards.

Anastomotic strength may be assessed both from the bursting pressure, which represents its resistance to intraluminal pressure, and from the breaking strength, which reflects its ability to withstand longitudinal forces.

Figure 2 depicts the outcome of all measurements of anastomotic bursting pressure performed at 3 days after operation. At this time the bursting site was always within the anastomotic area. In altogether six (out of 80) anastomoses the bursting pressure could not be measured because of technical problems. Neither 5-FU nor interferon-α administration led to a significant change in bursting pressure. However, the mean bursting pressure of ileal anastomoses in the 5-FU/interferon-α group (44±7 mmHg) was significantly ($P=0.0104$) lower than that in the control group (70±20 mmHg). In addition, it was also significantly reduced with respect to the 5-FU group ($P=0.0037$) and the interferon-α group ($P=0.0062$). In colon, these differences did not reach statistical significance. At 7 days after operation the bursting site was always outside the suture line and therefore the bursting pressures measured (data not shown) did not reflect actual wound strength.

When measuring the breaking strength (Figure 3) the breaking site was invariably located within the wound area. At 3 days after operation no differences were found between groups, but after 7 days the breaking strength of both ileal and colonic anastomoses was significantly lower in the 5-FU/interferon-α group than in the control group. Also, anastomotic breaking strength in the colon was reduced in the interferon-α group.

The hydroxyproline content in 5 mm segments containing the anastomosis was quantitated as a measure of wound collagen levels (Figure 4). No differences between the control group and the experimental groups were observed. The hydroxyproline content increased similarly from 3 to 7 days after operation independent of medication. Likewise, no differences were found for hydroxyproline concentrations. Mean hydroxyproline concentrations were 7.0±1.3 and 9.6±1.8 $\mu g\ mg^{-1}$ dry weight in 3- and 7-day-old ileal anastomoses respectively; corresponding values in colonic

**Figure 3** Anastomotic breaking strength. Bars represent mean values ($n=9$ or 10)+s.d. 1, control group; 2, 5-FU group; 3, interferon-α group; 4, 5-FU/interferon-α group. *Significantly ($P<0.033$, see Materials and methods) different from control group.

**Figure 2** Anastomotic bursting pressure after 3 days. Points represent measurements in individual animals with bars indicating mean values. *Significantly ($P<0.033$, see Materials and methods) different from control group.

**Figure 4** Anastomotic hydroxyproline content. Bars represent mean values ($n=10$)+s.d. 1, control group; 2, 5-FU group; 3, interferon-α group; 4, 5-FU/interferon-α group.

anastomoses were 9.7±0.7 and 13.8±1.9 μg $mg^{-1}$ dry weight. Similar values were measured in the experimental groups (data not shown).

The collagen synthetic capacity, measured *ex vivo* in tissue explants, was assayed in control segments removed at operation and in 3-day-old anastomotic tissue from the same rats. Table I shows that in the control group collagen synthetic capacity, expressed on the basis of DNA, wet weight or protein, was strongly increased in wound tissue. This increase was significantly ($P=0.0313$) higher in ileum than in colon. The fact that the percentage relative collagen synthesis was also elevated indicates that collagen synthesis was stimulated to a larger extent than the synthesis of non-collagenous proteins. Figure 5 depicts the anastomotic collagen (and non-collagenous protein) synthetic capacity, calculated on the basis of DNA content, for the various groups. Treatment with 5-FU significantly reduced collagen synthetic capacity without affecting the production of non-collagenous protein. In contrast, treatment with interferon-α did not appear to affect these processes to any substantial degree since no significant differences were seen between the interferon-α group and the control group or between the 5-FU/interferon-α group and the 5-FU group. Similar results were obtained if the collagen synthetic capacity was expressed on the basis of wet weight or protein content (data not shown).

### Discussion

The continuing search for effective adjuvant therapy after resection of colorectal carcinoma has resulted in a growing interest in the efficacy of both immediate post-operative regional chemotherapy (see Påhlman, 1995) and the combination of 5-FU with interferon-α as the cytostatic drugs of choice (see Grem *et al.*, 1995). The present results indicate that caution should be exerted in the use of interferon-α as an adjunct to 5-FU therapy in the early post-operative period since such treatment might constitute a threat to undisturbed anastomotic healing. The combination of interferon-α and 5-FU, administered intraperitoneally on the day of operation and the first two post-operative days, reduces the development of anastomotic strength during the first week after its construction. Administration of 5-FU alone has no significant deleterious effect on wound strength, but interferon-α in itself significantly lowers strength below control values in 7-day-old colonic anastomoses.

The wound healing process is characterised by massive cell migration and proliferation. Cytostatic drugs are by nature anti-proliferative and may therefore be expected to interfere with wound healing. Indeed, 5-FU administered daily from the day of operation onwards until sacrifice after 7 days severely impairs anastomotic healing in the rat intestine (Graf *et al.*, 1992; de Waard *et al.*, 1995*b*). In earlier experiments, we tried to mitigate this negative effect by concomitant administration of either interleukin 2 or granulocyte–macrophage colony-stimulating factor, but 5-FU impaired repair was not essentially altered by either cytokine. On the other hand, retinol significantly promoted 5-FU suppressed anastomotic healing (de Waard *et al.*, 1995*a*). Also, we reported before that the negative effect is limited if 5-FU is given only three times (de Waard *et al.*, 1993, 1995*a*). Since we expected any additional effect of interferon-α to be observed more easily under the latter conditions, we limited drug administration to the first 3 days.

**Table I** Increase in anastomotic collagen synthetic capacity 3 days after operation

| | *Control segment* | *Anastomosis* | *Ratio* |
|---|---|---|---|
| Ileum | | | |
| D.p.m. $\mu g^{-1}$ DNA | 41±6 | 194±30 | 4.9±1.2 |
| D.p.m. $mg^{-1}$ wet weight | 85±24 | 595±72 | 7.6±2.2 |
| D.p.m. $mg^{-1}$ protein | 2578±654 | 14786±1953 | 6.0±1.4 |
| RCS (%) | 0.47±0.08 | 1.02±0.18 | 2.2±0.5 |
| Colon | | | |
| D.p.m. $\mu g^{-1}$ DNA | 86±18 | 221±62 | 2.6±0.9 |
| D.p.m. $mg^{-1}$ wet weight | 305±45 | 773±150 | 2.6±0.7 |
| D.p.m. $mg^{-1}$ protein | 7317±1019 | 17454±3214 | 2.4±0.4 |
| RCS (%) | 1.12±0.12 | 1.72±0.32 | 1.5±0.2 |

Explants from control segments, collected at operation, and anastomotic tissue, collected 3 days after operation, were incubated for 3 h with 4.5 μCi of [$^3$H]proline. Collagen synthesis is expressed as radioactivity in collagenase-digestible protein and as percentage relative collagen synthesis (RCS). Data represent average values (+s.d.) from six animals.

**Figure 5** Anastomotic collagen synthetic capacity. Bars represent mean values ($n=6$)+s.d. of *ex vivo* synthesis of collagen (**a**, expressed as radioactivity in collagenase-digestible protein; **c**, expressed as percentage relative collagen synthesis, RCS) and other proteins (**b**, expressed as radioactivity in non-collagenous protein). 1, control group; 2, 5-FU group; 3, interferon-α group; 4, 5-FU/interferon-α group. Differences between groups are considered significant at $P<0.017$ (see Materials and methods) and nearly significant (symbols in brackets) at $P<0.033$. *, significant *vs* control group; $, significant *vs* 5-FU group; #, significant *vs* interferon-α group.

So far, little is clear about the effects of interferons on wound healing. Interferon-$\gamma$, delivered intraperitoneally through an osmotic pump (Granstein *et al.*, 1989) or injected subcutaneously (Miles *et al.*, 1994), has been found to delay skin repair in mice. In the latter study, wound disruption strength was reduced significantly at a dose of $7 \times 10^4$ U $kg^{-1}$ body weight and higher. Two experiments, with opposite results, have been reported on the effects of interferon-$\alpha/\beta$ on cutaneous healing in rodents. Intramuscular injection of interferon-$\alpha/\beta$ or intraperitoneal administration of polyinosinic-polycytidylic acid, a potent inducer of interferon, seemingly enhances repair (Bhartiya *et al.*, 1992), while local subcutaneous injection of interferon-$\alpha/\beta$ beneath the wound actually inhibits repair (Stout *et al.*, 1993). In these studies, evaluation of repair was solely on the basis of macroscopic or histological parameters; functional parameters, like wound strength, were not reported.

The present study is the first effort to investigate the effect of purified interferon-$\alpha$ on wound strength. Rat recombinant interferon-$\alpha$ was given in a daily dose of $2 \times 10^4$ U $kg^{-1}$ body weight. This dose is more than sufficient to protect rats against a lethal pseudorabies virus (PH van der Meide, personal communication), but is substantially lower than the doses of interferon employed in the studies mentioned above. Still, daily doses of this relatively low dose of interferon-$\alpha$, administered on the first three post-operative days, significantly reduce anastomotic breaking strength in the colon 7 days after operation. The results of treatment with the combination of 5-FU and interferon-$\alpha$ are probably of more immediate interest in terms of potential treatment of patients with colorectal cancer. It seems clear that addition of interferon-$\alpha$ to a regimen of 5-FU, which in itself does not affect anastomotic strength, may lead to a significant and substantial reduction in anastomotic strength during the first week of healing. In this period, where clinically most leakages occur, the strength of the anastomosed segment is relatively low as compared with the strength of the uninjured intestine: any further reduction constitutes a threat to anastomotic integrity and increases the chances for anastomotic dehiscence. Thus, these data should be treated as a warning that clinical application of 5-FU plus interferon-$\alpha$ immediately after anastomotic construction, although possibly beneficial with respect to adjuvant effectivity, may result in undesirable (side-) effects with respect to wound repair.

It remains to be determined how exactly interferon-$\alpha$ interferes with the healing sequence. The strength of both the uninjured and the sutured bowel wall depends to a large extent on collagen and anastomotic construction leads very quickly to an increased collagen synthetic capacity within the wound area (Martens and Hendriks, 1991). Interferons are known to be able to inhibit collagen synthesis (Granstein *et al.*, 1990). This suppressive effect is well established by *in vitro* experiments with fibroblasts (Jimenez *et al.*, 1984; Duncan and Berman, 1985), which are the primary producers of extracellular matrix in the healing wound. Indeed, histological examination of interferon-$\gamma$-treated wounds indicates reduced accumulation of collagen (Granstein *et al.*, 1989; Miles *et al.*, 1994). However, our data show that treatment with interferon-$\alpha$ alone does not lead to lowering of either *ex vivo* collagen synthetic capacity in anastomotic explants or hydroxyproline accumulation in anastomotic segments. Such an effect is indeed observed after 5-FU treatment, but again addition of interferon-$\alpha$ does not lead to further reduction. This lack of effect of interferon-$\alpha$ may be explained by the relatively low doses we used. Alternatively, it could be that interferon-$\alpha$ has less effect on matrix production than the other interferons. Experiments with isolated cells show this to be true for fibroblast collagen synthesis, both on the protein (Jimenez *et al.*, 1984; Duncan and Berman, 1985) and the mRNA level (Duncan *et al.*, 1995).

Anastomotic strength will also be affected by degradation of the existing matrix anchoring the sutures. The methodology used to measure the hydroxyproline content in anastomotic segments, which necessarily contain uninjured tissue next to the actual wound area, does not allow the detection of very localised loss of collagen. It may be that interferon-$\alpha$ increases collagenase expression (Duncan and Berman, 1989; Hujanen *et al.*, 1994), although macrophage metalloproteinase production appears to be inhibited by interferon-$\gamma$ (Wahl and Corcoran, 1993).

Finally, one could speculate that interferon-$\alpha$ interferes with healing by the inhibition of proliferation, either directly or by biochemical modulation of 5-FU metabolism. Interferons are growth inhibitors for a variety of normal and transformed cell lines (see Mallat *et al.*, 1995). The impairment of cutaneous healing by interferon-$\alpha/\beta$ is thought to be caused primarily by inhibition of proliferation of all cell types involved in wound repair (Stout *et al.*, 1993). More specifically, interferon-$\alpha$ is known to inhibit endothelial cell proliferation and thereby angiogenesis (Folkman, 1995), processes inherent to successful repair.

Whatever the mechanism(s) responsible, our data clearly indicate that administration of interferon-$\alpha$ in the perioperative period may be detrimental to anastomotic repair and that its use in immediate post-operative adjuvant therapy, as a means to enhance 5-FU activity, should be approached with caution.

**Acknowledgement**
The authors are grateful to Dr PH van der Meide (Institute of Applied Radiobiology and Immunology TNO, Rijswijk, The Netherlands) for his gift of rat recombinant interferon-$\alpha$.

### References

BHARTIYA D, SKLARSH JW AND MAHESHWARI RK. (1992). Enhanced wound healing in animal models by interferon and interferon inducer. *J. Cell. Physiol.*, **150,** 312–319.

BURTON KA. (1956). A study of the conditions and mechanisms of the diphenylamine reaction for the colorimetric estimation of deoxyribonucleic acid. *Biochem. J.*, **62,** 15–23.

CUNLIFFE WJ AND SUGARBAKER PH. (1989). Gastrointestinal malignancy: rationale for adjuvant therapy using early post-operative intraperitoneal chemotherapy. *Br. J. Surg.*, **76,** 1082–1090.

DE ROY VAN ZUIDEWIJN DBW, HENDRIKS T, WOBBES T AND DE BOER HHM. (1991). Intraperitoneal cytostatics impair healing of experimental intestinal anastomoses. *Br. J. Cancer*, **63,** 937–941.

DE WAARD JWD, WOBBES T AND HENDRIKS T. (1993). Early post-operative 5-fluorouracil does not affect the healing of experimental intestinal anastomoses. *Int. J. Colorect. Dis.*, **8,** 175–178.

DE WAARD JWD, WOBBES T, VAN DER LINDEN CJ AND HENDRIKS T. (1995*a*). Retinol may promote 5-fluorouracil-suppressed healing of experimental intestinal anastomoses. *Arch. Surg.*, **130,** 959–965.

DE WAARD JWD, WOBBES T, DE MAN BM, VAN DER LINDEN CJ AND HENDRIKS T. (1995*b*). Post-operative levamisole may compromise early healing of experimental intestinal anastomoses. *Br. J. Cancer*, **72,** 456–460.

DUNCAN MR AND BERMAN B. (1985). $\gamma$ Interferon is the lymphokine and $\beta$ interferon the monokine responsible for inhibition of fibroblast collagen production and late but not early fibroblast proliferation. *J. Exp. Med.*, **162,** 516–527.

DUNCAN MR AND BERMAN B. (1989). Differential regulation of glycosaminoglycan, fibronectin, and collagenase production in cultured human dermal fibroblasts by interferon-alpha, -beta, and -gamma. *Arch. Dermatol. Res.*, **281,** 11–18.

DUNCAN MR, HASAN A AND BERMAN B. (1995). Pentoxifylline, pentifylline, and interferons decrease type I and III procollagen mRNA levels in dermal fibroblasts: evidence for mediation by nuclear factor 1 down regulation. *J. Invest. Dermatol.*, **104**, 282-286.

FOLKMAN J. (1995). Clinical application of research on angiogenesis. *N. Engl. J. Med.*, **333**, 1757-1763.

GRAF W, WEIBER S, GLIMELIUS B, JIBORN H, PÅHLMAN L AND ZEDERFELDT B. (1992). Influence of 5-fluorouracil and folinic acid on colonic healing: an experimental study in the rat. *Br. J. Surg.*, **79**, 825-828.

GRANSTEIN RD, DEAK MR, JACQUES SL, MARGOLIS RJ, FLOTTE TJ, WHITAKER D, LONG FH AND AMENTO EA. (1989). The systemic administration of gamma interferon inhibits collagen synthesis and acute inflammation in a murine skin wounding model. *J. Invest. Dermatol.*, **93**, 18-27.

GRANSTEIN RD, FLOTTE TJ AND AMENTO EP. (1990). Interferons and collagen production. *J. Invest. Dermatol.*, **95**, 75S-80S.

GREM JL, MCATEE N, MURPHY RF, BALIS FM, STEINBERG SM, HAMILTON JM, SORENSEN JM, SARTOR O, KRAMER BS, GOLDSTEIN LJ, GAY LM, CAUBO KM, GOLDSPIEL B AND ALLEGRA CJ. (1991). A pilot study of interferon alpha-2a in combination with fluorouracil plus high-dose leucovorin in metastatic gastrointestinal carcinoma. *J. Clin. Oncol.*, **9**, 1811-1820.

GREM JL, VAN GROENINGEN CJ, ISMAIL AA, JOHNSTON PG, ALEXANDER HR AND ALLEGRA CJ. (1995). The role of interferon-$\alpha$ as a modulator of fluorouracil and leucovorin. *Eur. J. Cancer*, **31A**, 1316-1320.

HARRIS DT AND MASTRANGELO MJ. (1991). Theory and application of early systemic therapy. *Semin. Oncol.*, **18**, 493-503.

HESP FLEM, HENDRIKS T, LUBBERS EJC AND DE BOER HHM. (1984). Wound healing in the intestinal wall: a comparison between experimental ileal and colonic anastomoses. *Dis. Colon Rectum*, **27**, 99-104.

HUJANEN ES, VAISANEN A, ZHENG A, TRYGGVASON K AND TURPEENNIEMI-HUJANEN T. (1994). Modulation of $M_r$ 72,000 and $M_r$ 92,000 type-IV collagenase (gelatinase A and B) gene expression by interferons alpha and gamma in human melanoma. *Int. J. Cancer*, **58**, 582-586.

JIMENEZ SA, FREUNDLICH B AND ROSENBLOOM J. (1984). Selective inhibition of human diploid fibroblast collagen synthesis by interferons. *J. Clin. Invest.*, **74**, 1112-1116.

KEMENY N, LOKICH JJ, ANDERSON N AND AHLGREN J. (1993). Recent advances in the treatment of advanced colorectal cancer. *Cancer*, **71**, 9-18.

MALLAT A, PREAUX AM, BLAZEJEWSKI S, ROSENBAUM J, DHUMEAUX D AND MAVIER P. (1995). Interferon alpha and gamma inhibit proliferation and collagen synthesis of human ito cells in culture. *Hepatology*, **21**, 1003-1010.

MARTENS MFWC AND HENDRIKS T. (1991). Postoperative changes in collagen synthesis in intestinal anastomoses of the rat. Differences between small and large bowel. *Gut*, **32**, 1482-1487.

MARTENS MFWC, DE MAN BM, HENDRIKS T AND GORIS RJA. (1992). Collagen synthesis througout the uninjured and anastomosed intestinal wall. *Am. J. Surg.*, **164**, 354-360.

MILES RH, PAXTON TP, ZACHEIS D, DRIES DJ AND GAMELLI RL. (1994). Systemic administration of interferon-$\gamma$ impairs wound healing. *J. Surg. Res.*, **56**, 288-294.

MOERTEL CG. (1994). Chemotherapy for colorectal cancer. *N. Engl. J. Med.*, **330**, 1136-1142.

PÅHLMAN L. (1995). Open trials in colorectal cancer. *Eur. J. Surg. Oncol.* **21**, 347-351.

PAZDUR R. (1991). Fluorouracil and recombinant interferon alfa-2a in advanced gastrointestinal neoplasms. *Br. J. Haematol.*, **79**, 56-59.

PETERKOFSKY B, CHOIKIER M AND BATEMAN J. (1981). Determination of collagen synthesis in tissue and cell culture systems. In *Immunochemistry of the Extracellular Matrix*, Vol. 2, Furthmayer H. (ed.) pp. 19-47. CRC Press: Boca Raton, FL.

SEN GC AND LENGYEL P. (1992). The interferon system. A bird's eye view of its biochemistry. *J. Biol. Chem.*, **267**, 5017-5020.

SMITH PK, KROHN RI, HERMANSON GT, MALLIA AK, GARTNER FH, PROVENZANO MD, FUJIMOTO EK, GOEKE NM, OLSON BJ AND KLENK DC. (1985). Measurement of protein using bicinchoninic acid. *Anal. Biochem.*, **150**, 76-85.

STOUT AJ, GRESSER I AND THOMPSON WD. (1993). Inhibition of wound healing in mice by local interferon $\alpha/\beta$ injection. *Int. J. Exp. Pathol.*, **74**, 79-85.

VAN DER MEIDE PH, DIJKEMA R, CASPERS M, VIJVERBERG K AND SCHELLEKENS H. (1986). Cloning, expression, and purification of rat IFN-$\alpha$1. *Methods Enzymol.*, **119**, 441-453.

WADLER S, LEMBERSKY B, ATKINS M, KIRKWOOD J AND PETRELLI N. (1991). Phase II trial of fluorouracil and recombinant interferon alpha-2a in patients with advanced colorectal carcinoma: an Eastern Cooperative Oncology Group study. *J. Clin. Oncol.*, **9**, 1806-1810.

WAHL L AND CORCORAN ML. (1993). Regulation of monocyte/macrophage metalloproteinase production by cytokines. *J. Periodontol.*, **64**, 467-473.